# Activation of CRH receptor type 1 expressed on glutamatergic neurons increases excitability of CA1 pyramidal neurons by the modulation of voltage-gated ion channels

**DOI:** 10.3389/fncel.2013.00091

**Published:** 2013-07-19

**Authors:** Stephan Kratzer, Corinna Mattusch, Michael W. Metzger, Nina Dedic, Michael Noll-Hussong, Karl W. Kafitz, Matthias Eder, Jan M. Deussing, Florian Holsboer, Eberhard Kochs, Gerhard Rammes

**Affiliations:** ^1^Department of Anesthesiology, Klinikum Rechts der Isar der Technischen Universität MünchenMunich, Germany; ^2^RG Molecular Neurogenetics, Max Planck Institute of PsychiatryMunich, Germany; ^3^Clinical Cooperation Group Molecular Neurogenetics, Institute of Developmental Genetics, Helmholtz Center MunichNeuherberg, Germany; ^4^Department of Psychosomatic Medicine and Psychotherapy, University of UlmUlm, Germany; ^5^Faculty of Mathematics and Natural Sciences, Institute of Neurobiology, Heinrich Heine University DüsseldorfDüsseldorf, Germany; ^6^RG Neuronal Network Dynamics, Max Planck Institute of PsychiatryMunich, Germany; ^7^Max Planck Institute of PsychiatryMunich, Germany

**Keywords:** CRH, CRH receptor, neuronal excitability, potassium channels, protein kinases

## Abstract

Corticotropin-releasing hormone (CRH) plays an important role in a substantial number of patients with stress-related mental disorders, such as anxiety disorders and depression. CRH has been shown to increase neuronal excitability in the hippocampus, but the underlying mechanisms are poorly understood. The effects of CRH on neuronal excitability were investigated in acute hippocampal brain slices. Population spikes (PS) and field excitatory postsynaptic potentials (fEPSP) were evoked by stimulating Schaffer-collaterals and recorded simultaneously from the somatic and dendritic region of CA1 pyramidal neurons. CRH was found to increase PS amplitudes (mean ± Standard error of the mean; 231.8 ± 31.2% of control; *n* = 10) while neither affecting fEPSPs (104.3 ± 4.2%; *n* = 10) nor long-term potentiation (LTP). However, when Schaffer-collaterals were excited via action potentials (APs) generated by stimulation of CA3 pyramidal neurons, CRH increased fEPSP amplitudes (119.8 ± 3.6%; *n* = 8) and the magnitude of LTP in the CA1 region. Experiments in slices from transgenic mice revealed that the effect on PS amplitude is mediated exclusively by CRH receptor 1 (CRHR1) expressed on glutamatergic neurons. The effects of CRH on PS were dependent on phosphatase-2B, L- and T-type calcium channels and voltage-gated potassium channels but independent on intracellular Ca^2+^-elevation. In patch-clamp experiments, CRH increased the frequency and decay times of APs and decreased currents through A-type and delayed-rectifier potassium channels. These results suggest that CRH does not affect synaptic transmission *per se*, but modulates voltage-gated ion currents important for the generation of APs and hence elevates by this route overall neuronal activity.

## Introduction

The neuropeptide corticotropin-releasing hormone (CRH) is the main regulator for the hypothalamic-pituitary-adrenal (HPA) axis and plays a central role in the mammalian adaptive response to stress (Bonfiglio et al., 2011). Besides inducing synthesis and release of adrenocorticotropic hormone (ACTH) secretion from the anterior pituitary, CRH exerts a wide spectrum of behavioral and autonomic effects in the central nervous system (Hillhouse and Grammatopoulos, 2006).

Increased expression and release of CRH not only results in activation of the neuroendocrine HPA-axis, but via projections from the hypothalamus and on-site production at other brain locations also in behavioral changes important to adapt to stress. Once stressors persist the continuous hyperactivity of CRH neurons can lead to anxiety- and depression-like behavior in animals. Increased concentrations of CRH were found in the cerebrospinal fluid (CSF) of a subset of patients with depression (Nemeroff et al., 1984; Holsboer, 2000) and also a number of endocrine function tests (Holsboer, 2000) were in agreement with elevations of central CRH in many severely depressed patients. Due to these and other findings CRH and its two receptors (CRHR1 and CRHR2) became the focus of studies of causality of mood disorders and the search for pharmacological targets.

The hippocampal formation (HF) plays a key role in memory storage and learning, however, in recent years it has also received significant attention in mood disorder research. The release of endogenous hippocampal CRH can be induced by physiological stress (Chen et al., 2004). Direct injection of CRH into the dentate gyrus enhanced memory retention in rats (Lee et al., 1993), whereas injection into the dorsal hippocampus improved context- and tone-dependent fear conditioning (Radulovic et al., 1999). In patients with major depressive disorder, imaging studies showed structural and functional alterations in the HF (Campbell and MacQueen, 2004). CRH-immunoreactive cells can be found in the hippocampus of rats at P1 and the number of these neurons increases until P18 (Chen et al., 2001). Most of the CRH-releasing cells are GABAergic neurons that build synapses on the somata of pyramidal neurons. This anatomical location provides them a significant influence on pyramidal cell activity and information flow (Korosi and Baram, 2008) since CRHR1 is discussed to be expressed on pyramidal neurons (Aguilera et al., 2004).

Within the mouse hippocampus, CRH has been shown to be synthesized in GABAergic neurons and mRNA encoding CRHR1 was found in the hippocampal subregions CA1 and CA3 in mice (Van Pett et al., 2000).

The effect of CRH on neuronal activity within the HF is still not entirely understood. Previous studies showed a long lasting enhancement of synaptic efficacy in the hippocampus (Wang et al., 1998), an enhancement of CA1 population spikes (PS) and an increase in firing frequency of rat hippocampal neurons (Aldenhoff et al., 1983; Blank et al., 2002; Sillaber et al., 2002). Another study using the voltage-sensitive dye imaging technique in acute slices described a CRHR1 dependent increase in neuronal activity propagation through the HF (von Wolff et al., 2011). The exact mechanism how CRH mediates these versatile actions is still unclear. In this explorative study we investigated the influence of CRH on neuronal excitability of CA1 principal neurons in rodents to further elucidate the underlying mechanisms of the CRH action.

## Materials and methods

### Animals

The experimental protocols were approved by the Ethical Committee on Animal Care and Use of the Government of Bavaria (Munich, Germany).

Mice lacking CRHR1 on glutamatergic and GABAergic neurons, respectively, have been described previously (Refojo et al., 2011). Briefly: Homozygous floxed CRHR1 mice (CRHR1^flox/flox^) were bred to Nex-Cre mice resulting in the F2 generation in CRHR1^flox/flox^ Nex-Cre mice (CRHR1^Glu-CKO^) lacking CRHR1 on glutamatergic neurons. Homozygous floxed CRHR1 mice (CRHR1^flox/flox^) to Dlx5/6-Cre mice resulting in the F2 generation in CRHR1^flox/flox^ Dlx5/6-Cre mice (CRHR1^GABA-CKO^) lacking CRHR1 on GABAergic neurons. As controls respective homozygous floxed littermates lacking Cre recombinase were used (CRHR1^Ctrl^). Both mouse lines are on a mixed 129S2/Sv × C57BL/6J genetic background.

Mice harboring a conditional Ca_V_1.2 allele (CaV1.2loxP) have been described previously (Seisenberger et al., 2000). Conditional knockout mice devoid of Ca_V_1.2 in the entire central nervous system were generated by breeding Ca_V_1.2loxP/loxP to Ca_V_1.2± Nestin-Cre mice, resulting in CNS specific Ca_V_1.2 knockout mice (Ca_V_1.2^CNS-CKO^; Ca_V_1.2−/loxP Nestin-Cre) and respective control mice (Ca_V_1.2^ctrl^; Ca_V_1.2+/loxP Nestin-Cre).

### Electrophysiology

Male CD1 or transgenic mice were anesthetized with isoflurane and after decapitation, brains were quickly transferred into ice-cold carbogenated (95% O_2_/5% CO_2_) artificial cerebrospinal fluid (aCSF), and sagittal hippocampal slices (350 μm) were cut using a vibratome (HM 650 V, Microm International, Walldorf, Germany). Afterwards, slices were allowed to recover for at least 1 h at 34°C before being transferred to the recording chamber where they were continuously superfused with aCSF at a rate of 5 ml/min. The aCSF contained (in mM): NaCl, 124; KCl, 3; NaHCO_3_, 26; CaCl_2_, 2; MgSO_4_, 1; D-glucose, 25; NaH_2_PO_4_, 1.25, and was bubbled with carbogen (95% O_2_/5% CO_2_, final pH 7.3). CRH (rat/human; 125 nM final concentration, Sigma-Aldrich, Germany) was bath applied. All experiments were performed at room temperature (20–24°C).

Extracellular recordings in slices from mice (p 42–56) were performed using glass microelectrodes (2–3 MΩ) filled with aCSF. For recording of field excitatory postsynaptic potentials (fEPSPs) the electrode was placed in the CA1 stratum radiatum and for measurement of PS electrodes were placed in the CA1 stratum pyramidale. PS and fEPSPs were evoked by electrical stimulation of the Schaffer-collaterals (SC) with a bipolar tungsten electrode (SC-stim-fEPSPs; simulations intensities between 1 and 20 Volts for 50 μs). In the majority of the experiments, fEPSPs and PS were recorded simultaneously. Stimulation intensities were set to record SC-stim-fEPSPs without PS in CA1 stratum radiatum for one electrode and to record PS in CA1 stratum pyramidale for the other electrode. In a subset of experiments, the stimulation electrode was positioned in the CA3 pyramidal layer to stimulate the somata of CA3 pyramidal neurons and fEPSPs were recorded in CA1 stratum radiatum (CA3-stim-fEPSPs). This ensures the inclusion of an AP generation site within the recorded circuit and the transmitter release depends on somatic action potential (AP) initiation and axonal signal propagation. The influence of CRH on hippocampal long-term potentiation (LTP) of SC-stim-fEPSPs and CA3-stim-fEPSPs was investigated in another subset of experiments. For that purpose, a high-frequency stimulation (HFS; 100 Hz/1 s) train was applied on either the SC or the CA3 stratum pyramidale.

Whole cell patch-clamp recordings in slices from mice (p 28–42) were obtained from CA1 pyramidal neurons using infrared videomicroscopy (Dodt et al., 1999). The recording pipettes (resistance between 4.5 and 5 MΩ) were filled with (in mM) K-D-gluconate, 130; KCl, 5; EGTA, 0.5; Mg-ATP, 2; HEPES, 10; D-glucose, 5. Currents and potentials were recorded by a discontinuous voltage clamp amplifier (SEC 10, NPI electronics, Tamm, Germany) in the voltage-clamp mode (switching frequencies 40–43 kHz, 25% duty cycle) and bridge mode, respectively.

Voltage-clamp experiments were performed at a holding potential of −50 mV. For the measurement of potassium currents, cells were stepwise depolarized from the holding potential to +40 mV in 10 mV steps. In adult neurons potassium currents are composed of a rapidly inactivating A current (I_A_), a delayed rectifier current (I_K_) and a slowly inactivating K^+^ current (I_S_). Delivery of a short hyperpolarizing step to −100 mV preceding the depolarization removes a substantial portion of the inactivation from the fast I_A_ without de-inactivating I_S_ (Figure 6B). Depolarization from a more positive potential (−50 mV) activates only I_K_ as I_A_ is inactivated (Figure 6A). By graphical subtraction of currents resulting from voltage steps with and without the preceding hyperpolarizing step, I_A_ currents can be analyzed (Figure 6C). Sodium currents were measured by depolarization of CA1 neurons from a holding potential of −70 to −20 mV in 2 mV steps. For sodium current recordings, the pipettes were filled with (in mM) CsCl, 75; NaF, 75; MgCl_2_, 2; HEPES, 10; EGTA, 2.5; Na_2_ATP, 3 and slices were perfused with aCSF containing (in MM) NaCl, 80; Tetramethylammonium chloride, 45; KCl, 2.5; NaH_2_PO_4_, 1.25; D-glucose, 25; NaHCO_3_, 25; MgCl_2_, 1; CaCl, 2. For leak subtraction, the P/4 subtraction protocol was used.

For measuring the effect of CRH on L-type and T-type calcium channels in CA1 pyramidal cells, we substituted calcium in the extracellular solution with barium and thus recorded barium currents through calcium channels. The extracellular solution contained (in mM): NaCl 125, KCl 2.5, NaHCO_3_ 25, CaCl_2_ 2, BaCl_2_ 1, D-glucose 25, NaH_2_PO_4_ 1.25. Pipettes were filled with an intracellular solution containing (in mM): Cs-methane sulphonate 110, EGTA 0.5, MgCl_2_ 2, HEPES 10, D-glucose 5, Na_2_-phosphocreatine 14, Mg-ATP 5 and Na-GTP 0.3. L-type and T-type calcium channel currents were recorded by adding specific antagonists to the aCSF: ω-Conotoxin GVIA (1 μM) for N-type channels and ω-Agatoxin IVA (100 nM) for P/Q-type channels. Cells were held at −90 mV and a step to −40 mV was used to activate and inactivate the T current. After repolarization to −50 mV, a step to 0 mV was used to activate the L-current.

### Calcium imaging

In a subset of experiments, Ca^2+^ influx into CA1 pyramidal neurons upon electrical stimulation of the SC was measured by fluorometric calcium imaging. Therefore slices were loaded with the membrane soluble acetoxymethyl (AM) ester of oregon green (Invitrogen, Darmstadt, Germany) dissolved in DMSO + 20% Pluronic F-127 at a stock concentration of 5 mM. For neuron staining, the stock solution was diluted 1:10 with HEPES-aCSF (in mM: NaCl 125, KCl 3, HEPES 25, MgSO4 2, CaCl2 2, NaH2PO4 1.25) which resulted in a final dye concentration of 500 μM oregon green-AM. The dye was applied to the somata of CA1 neurons by ejecting it from a micropipette (open tip resistance 0.5 MΩ) with a mild pressure pulse. Cells were allowed to uptake the dye for 1 h before the beginning of recordings. Imaging was performed with a 60X-water immersion objective (numerical aperture 0.9; Olympus, Hamburg, Germany), and a filter block containing a 530/25 filter (band pass) and 510 dichroic mirror. A monochromator (Polychrome V; T.I.L.L. Photonics; Martinsried; Germany) was used for excitation with a wavelength of 494 nm and emission was collected at a maximum of 523 nm. For measurements of calcium-dependent changes in fluorescence, images were sampled during stimulation to the SC for 1.5 s at 20 Hz and recorded with a high speed CCD camera (Retiga-2000RV; Qimaging; Surrey; Canada). Extracellular PS recordings were performed in parallel to the imaging measurements for verifying the CRH effect. Data acquisition and offline data analysis were performed with TillVision software (T.I.L.L. Photonics). Baseline fluorescence before electrical stimulation (F0) and relative fluorescence changes (ΔF/F0) in defined regions of interest (ROIs) positioned to the cell somata in CA1 were measured throughout the image sequence. Changes in intracellular calcium levels are expressed as ΔF/F0. The time integral (area under the curve; AUC) was used to assess changes in total calcium.

### Analysis of data

Amplified currents, fEPSPs, and PSs were filtered (3 kHz), digitized (15 kHz) using a laboratory interface board (ITC-16, Instrutech Corp., USA) and stored to disk on a Power Macintosh G3 computer with the acquisition program Pulse, version 8.5 (Heka Electronic GmbH, Lambrecht, Germany). To measure PS amplitude the voltage difference between the sharp negative onset and the negative peak (a) and the one between the negative peak and the succeeding positive peak (b) was displayed, and the amplitude of the PS was calculated as (a + b)/2. All values were statistically analyzed using Student's paired *t*-test or Mann-Whitney-U test with a level of *p* < 0.05 required for significance. Averaged values are shown as mean ± SEM.

### Drugs

Drugs were applied via the superfusion system. Compounds used: All salts for the pipette solution and aCSF, K252a, H-89 dihydrochloride hydrate, H-7 dihydrochloride, Cyclosporine A and nifedipine were purchased from Sigma Aldrich (Munich, Germany). Oregon Green was obtained from Life Technologies GmbH (Darmstadt, Germany) and CRH (human/rat) from Bachem (Weil am Rhein, Germany).

## Results

**Figure 1:**
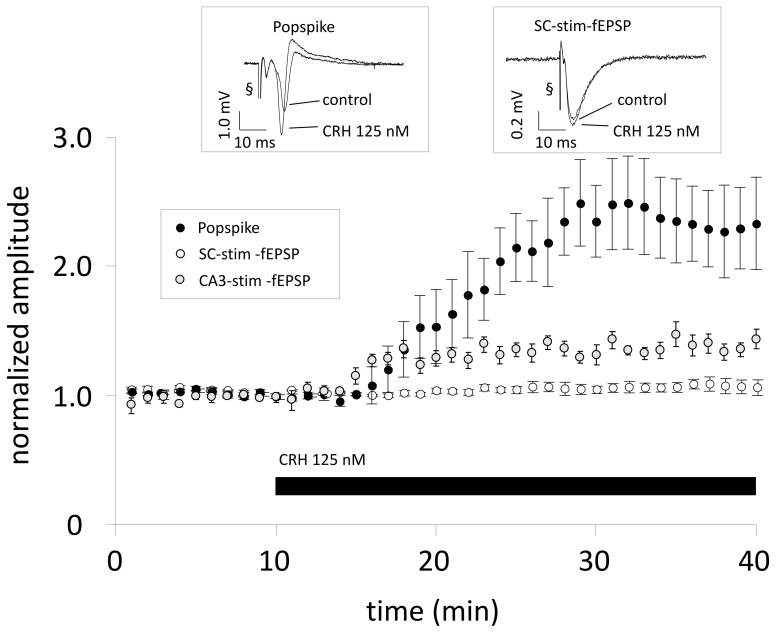
**When Schaffer-collaterals are electrically stimulated CRH increases the population spike (PS) amplitude without affecting field excitatory postsynaptic potentials (fEPSPs).** Acute sagittal hippocampal brain slices were obtained from male CD-1 mice (P30–P50). Dual recording was performed by positioning one electrode in the stratum radiatum of the CA1 region for monitoring fEPSPs (SC-stim-fEPSPs; white symbols) and the second electrode was positioned in the pyramidal layer for monitoring PS (black symbols). After 10 min of reaching a stable baseline, CRH (125 nM) was applied to the perfusion medium. CRH increases PS amplitudes to 231.8 ± 31.2% of baseline level (*n* = 10; *p* < 0.05) without affecting fEPSP slope (104.3 ± 4.2% of control; *n* = 10; *p* > 0.05). In a subset of experiments the stimulation electrode was positioned in the CA3 pyramidal layer and fEPSPs were recorded from the CA1 stratum radiatum (CA3-stim-fEPSPs; gray symbols). This stimulation technique allows the inclusion of an action potential generation site and, consequently, transmitter release at CA3-CA1 synapses is dependent on somatic action potential initiation and axonal signal propagation. Under these conditions, CRH (125 nM) significantly increased CA3-stim-fEPSP amplitudes to 119.8 ± 3.6% of control (*n* = 8; *p* < 0.05). Each data point represents the mean ± SEM of 4 consecutive fEPSP/PS amplitude measurements normalized to the last 5 min before CRH application. Insets show representative recording traces; § stimulation artifact. Black bar indicates presence of CRH.

To test whether the effect of CRH on PS amplitude also results in increased fEPSP amplitudes one recording electrode was positioned in the stratum radiatum and one electrode in the stratum pyramidale for parallel recording of fEPSPs (SC-stim-fEPSPs) and PS, evoked by orthodromic and antidromic electrical stimulation of the SC with two stimulation electrodes. In CA1 region, CRH (125 nM) strongly enhanced PS amplitude to 231.8 ± 31.2% (*n* = 10; *p* < 0.05; Figure 1) with no effect on fEPSP amplitude 104.3 ± 4.2% (*n* = 10; *p* > 0.05; Figure 1).

**Figure 2:**
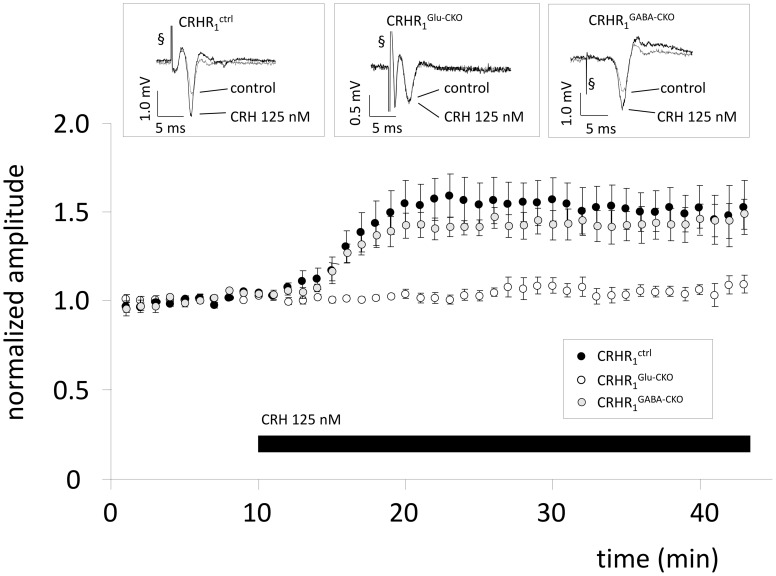
**The CRH-mediated effect depends on CRH receptor type 1 (CRHR1) expression on glutamatergic neurons.** Slices were prepared from conditional knock-out mice lacking CRHR1 either on glutamatergic neurons (CRHR1^Glu-CKO^) or on GABAergic neurons (CRHR1^GABA-CKO^). CRHR1^flox/flox^ mice littermates served as control.CRH increases PS amplitude in slices from CRHR1^ctrl^ to 154.7 ± 10.2% (*n* = 7; *p* < 0.05; black dots) and CRHR1^GABA-CKO^ (144.5 ± 9.5%; *n* = 7; *p* < 0.05; gray dots), but showed no effect in CA1 hippocampal neurons of CRHR1^Glu-CKO^ (98.2 ± 2.0%; *n* = 7; *p* < 0.05; white dots). Insets show representative recording traces. § stimulation artifact. Black bar represents presence of CRH.

To investigate whether the effect of CRH is dependent on CRHR1 expression on glutamatergic or GABAergic neurons, slices from conditional knock-out animals were used. In CRHR1^Ctrl^ mice CRH 125 nM increased the PS amplitude to 154.7 ± 10.2% of control (*n* = 7; *p* < 0.05; Figure 2). In CRHR1^Glu-CKO^ mice the effect of CRH was abolished (98.2 ± 2.0% of control; *n* = 7; *p* > 0.05; Figure 2) whereas in CRHR1^GABA-CKO^ mice CRH increased PS amplitudes to 144.5 ± 9.5% of control (*n* = 7; *p* > 0.05 vs. CRHR1^flox/flox^; Figure 2).

**Figure 3:**
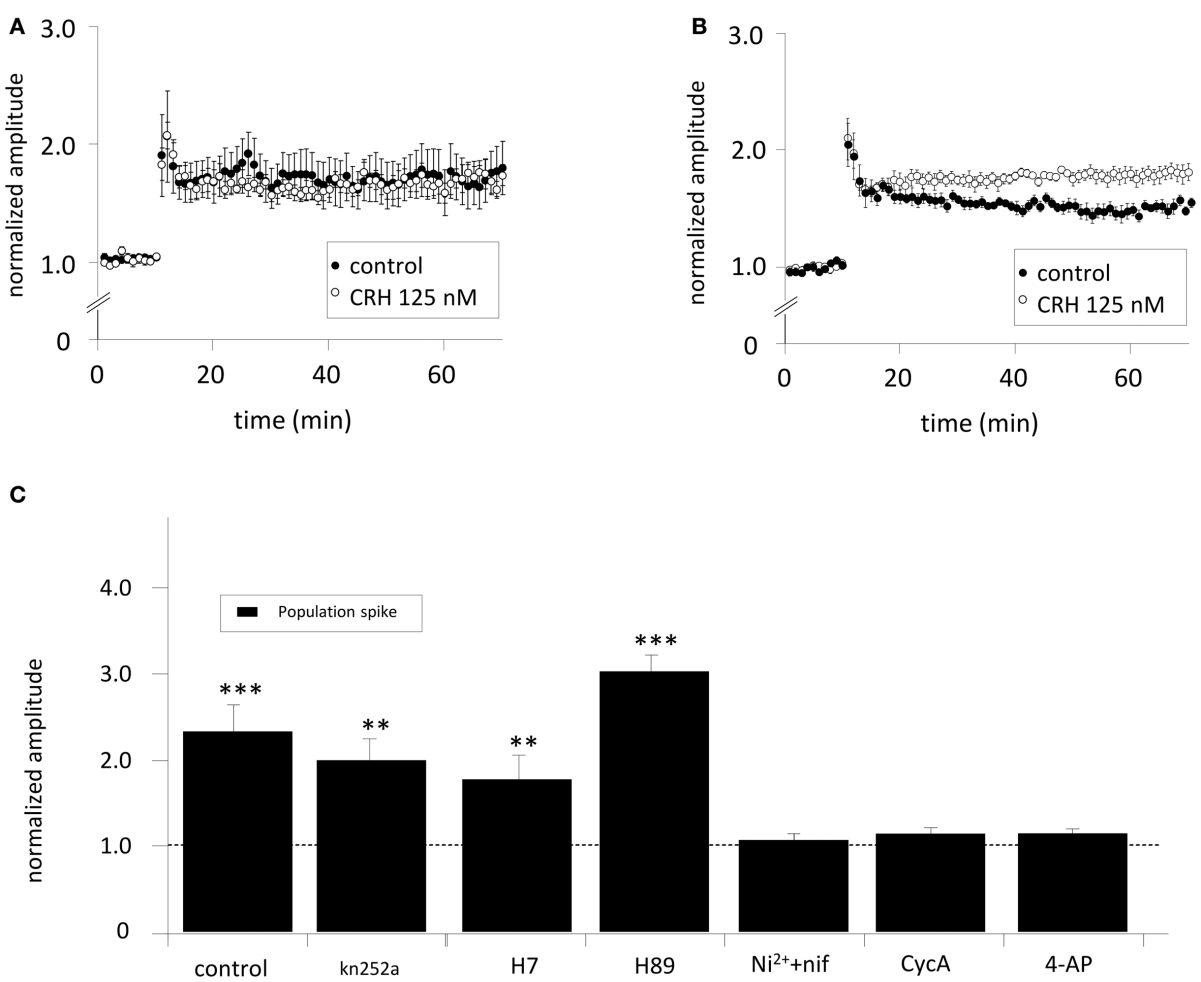
**(A)** The effect of CRH on CA1-LTP depends on the site of electrical stimulation. When SC-stim-fEPSPs were recorded, CRH did not change LTP induced by HFS (control: 173.3 ± 19.8 %; *n* = 5, in the presence of CRH: 171.3 ± 8.2%; *n* = 5, *p* > 0.05). However, when HFS was delivered to the CA3 stratum pyramidale, **(B)** CRH increased the potentiation of CA3-stim-fEPSPs from 154.8 ± 5.0% (*n* = 6) under control conditions to 180.6 ± 8.1% (*n* = 6; *p* < 0.05 for control vs. CRH). **(C)** The effect of CRH on the population spike amplitude is dependent on phosphatase 2B (PP-2B), voltage-gated potassium channels (K_V_) and T- and L-type calcium channels. PS were recorded from the CA1 pyramidal layer upon electrical stimulation of the Schaffer-collaterals. The inhibition of PKA, PKC, and CaMKII with Kn252a (500 nM), H7 (100 μM), and H89 (10 μM), respectively did not affect the CRH-mediated effect on PS amplitude. In the presence of the T- and L-type calcium channel antagonist nifedipine (nif) and Ni^2+^, respectively, CRH failed to enhance PS amplitudes (105.9 ± 7.6%; *n* = 6; *p* > 0.05). CRH did neither increase PS amplitudes when PP-2B was inhibited with cycA (113.1 ± 7.3%; *n* = 6; *p* > 0.05) nor when voltage-gated potassium channels were antagonized with 4-aminopyridine (4-AP; 113.5 ± 6.2%; *n* = 6; *p* > 0.05). ^**^p < 0.01; ^***^p < 0.001.

The CRH-mediated increase in PS amplitudes in CA1 might be mediated by increased somatic excitability by e.g., lowered threshold for AP (AP) generation. CRH increased CA3-stim-fEPSP amplitudes to 119.8 ± 3.6% of control (*n* = 8; *p* < 0.05; Figure 1). HFS applied to the SC induced LTP of SC-stim-fEPSPs to 173.3 ± 19.8% of control (*n* = 5; Figure 3A) which was not affected in the presence of CRH (171.3 ± 8.2%; *n* = 5; *p* > 0.05 for control vs. CRH Figure 3A). When the same HFS was delivered to the somatic region of CA3 pyramidal neurons, the magnitude of CA3-stim-LTP was significantly increased after the application of CRH (180.6 ± 8.1% vs. 154.8 ± 5.0%; *n* = 6; *p* < 0.05; Figure 3B).

To elucidate the neuronal mechanisms how CRH enhances the PS amplitude slices were incubated with either K252a (500 nM), H7 (100 μM), H89 (10 μM), Nifedipine (20 μM), and Ni^+^ (50 μM), Cyclosporine A (cycA; 50 μM) or 4-AP (100 μM) to block protein kinase A (PKA), protein kinase C (PKC), Ca^2+^/calmodulin-dependent protein kinase (CaMKII), T- or L-type Ca^2+^-channels, protein phosphatase 2B (PP-2B/ Calcineurin) and voltage-gated K^+^-channels (K_V_). The CRH-induced enhancement of PS amplitudes could be prevented by antagonising T- and L-type Ca^2+^-channels (96.2 ± 7.3%; *n* = 6; *p* > 0.05; Figure 3C), K_V_ (113.6 ± 6.2%; *n* = 6; *p* > 0.05; Figure 3C), and PP2B (98.4 ± 5.1%; *n* = 6; *p* > 0.05; Figure 3C). These results suggest a PP-2B and L-type calcium channel dependent mechanism. Application of the potassium channel antagonist 4-AP lead to increased PS amplitude and evoked epileptiform activity (repeated PS upon electrical stimulation). Previous to CRH application, stimulation intensity was reduced to basal levels eliciting only one single PS. Interestingly, the blockade of PKA and PKC activity with H7 induced a dramatic increase in PS amplitude *per se* to 424.6 ± 10.2% of control (*n* = 5; *p* < 0.05; data not shown). Previous to CRH application, PS amplitudes were reset to control.

**Figure 4:**
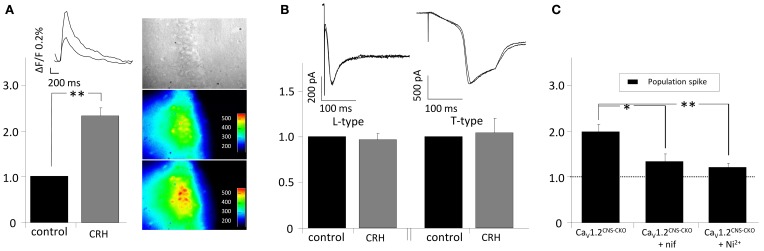
**CRH increases intracellular calcium influx in CA1 pyramidal neurons upon electrical stimulation of the Schaffer-collaterals. (A)** Cells were loaded with the calcium sensitive dye oregon green-AM. After 1 h of incubation, the SC were electrically stimulated every 15 s and calcium influx was recorded simultaneously (sampling rate 20 Hz; duration 1.5 s). In the presence of CRH the intracellular Ca^2+−^influx during synaptic activity increased to 231.7 ± 13.8% of control (*n* = 4; *p* < 0.05). Right insets: Videomicroscopic photograph of the CA1 region of the hippocampus and fluorescence recorded under control conditions (middle) and in the presence of 125 nM CRH (lower inset). **(B)** Barium currents through T- and L-type calcium channels were recorded in single-cell voltage-clamp recordings. CRH did neither increase L-currents (96.8 ± 6.4% of control; *n* = 6; *p* > 0.05) nor T-currents (103.8 ± 16.4% of control; *n* = 7; *p* > 0.05). **(C)** PS amplitudes recorded from Ca_V_1.2^CNS-CKO^ animals were increased to 197.3 ± 16.0% of control (*n* = 3; *p* < 0.05). The effect of CRH on PS amplitude in CNS-CKO animals was diminished in the presence of the calcium channel antagonists nifedipine (132.3 ± 10.0% of control; *n* = 4; *p* < 0.05) or Ni^2+^ (119.6 ± 5.9% of control; *n* = 5; *p* < 0.05). ^*^p < 0.05; ^**^p < 0.01.

Our experiments suggest an involvement of T- and L-type calcium channels in the CRH-mediated effect on PS amplitude. Thus, we monitored somatic intracellular Ca^2+^ influx in the presence of CRH. Application of CRH for 20 min did not change the cytosolic calcium level and hence ΔF/F (data not shown). However, when Ca^2+^ influx has been monitored during electrical stimulation of the SC, in the presence of CRH (125 nM) the AUC of the calcium signal was significantly increased to 231.7 ± 13.8% of control (*n* = 4; *p* < 0.05; Figure 4A). To further investigate the involvement of T- and L-type calcium channels in the CRH-mediated increase in PS amplitude, experiments in Ca_V_1.2 knockout animals were performed. In Ca_V_1.2 knockout animals, CRH (125 nM) increased PS amplitude to 197.3 ± 16.0% of control (*n* = 3; *p* < 0.05; Figure 4C). Thus, an involvement of Ca_V_1.2 seems rather unlikely. However, when either the calcium channel antagonists nifedipine or Ni^2+^ was added to the perfusate, the CRH-mediated enhancement in PS amplitude was strongly impaired (132.3 ± 10.0% of control in the presence of nif and 119.6 ± 5.9% of control in the presence of Ni^2+^; Figure 4C). Hence, the CRH-mediated increase in PS amplitude seems to be dependent on T- and L-type calcium channel function. Interestingly, experiments from single-cell recordings revealed, that CRH did neither affected currents through L-type (96.8 ± 6.4% of control; *n* = 6; *p* > 0.05; Figure 4B) nor through T-type (103.8 ± 16.4% of control; *n* = 7; *p* > 0.05; Figure 4B) voltage gated calcium channels. Additionally, the presence of the membrane-permeant calcium chelator BAPTA-acetoxymethyl ester (30 μM) did not diminish the CRH effect on PS amplitude (data not shown).

**Figure 5:**
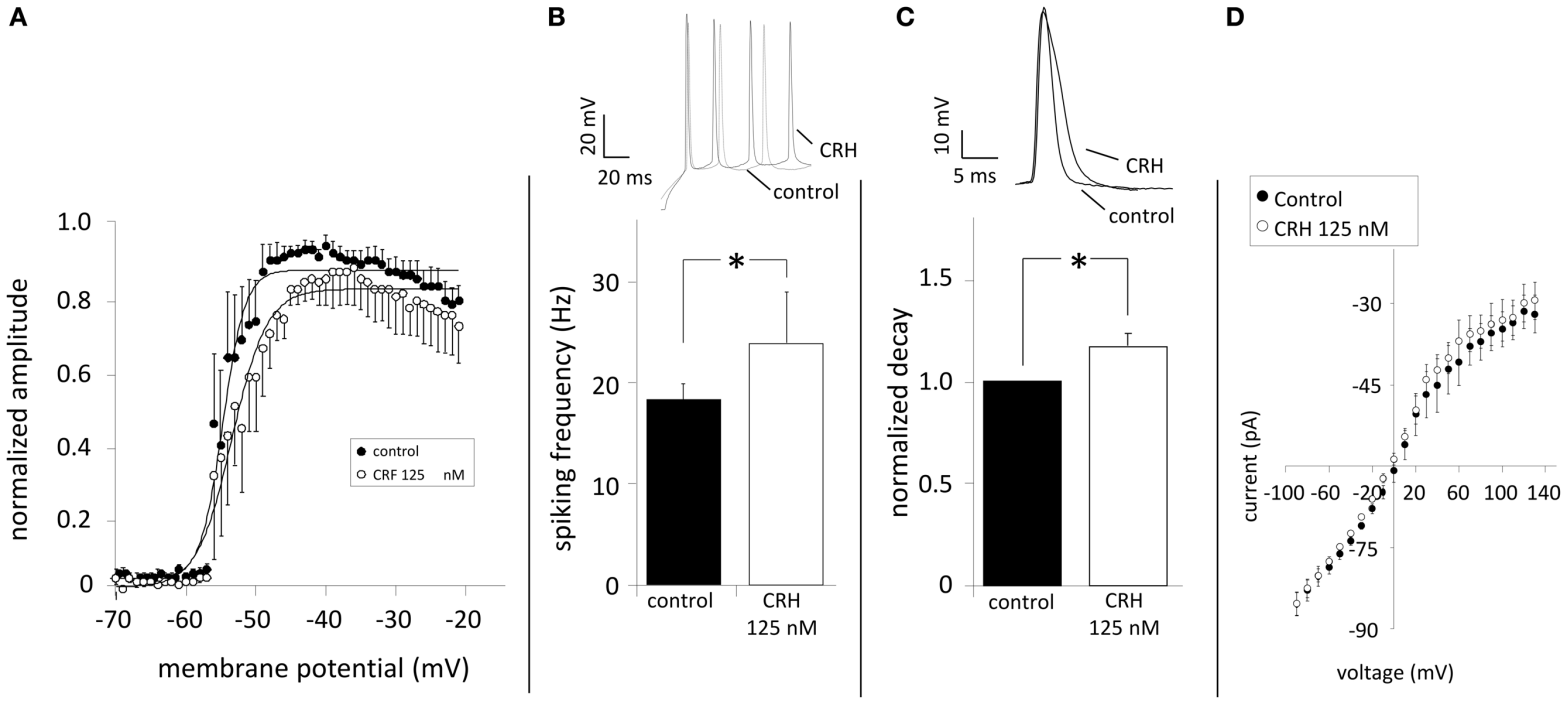
**CRH does not modulate Na_V_ channels but increases spiking frequency and prolongs action potentials.** Sodium currents were elicited by depolarizing the membrane from −70 to −20 mV (ΔU = 2 mV). **(A)** The normalized amplitude of Na^+^-channel current amplitudes was plotted against the membrane potential. Na_V_ channels showed half-maximum activation (V_1/2_) of 57.4 ± 0.2 mV (*n* = 7) under control conditions. In the presence of CRH (125 nM) V_1/2_ was not significantly different (52.8 ± 0.3; *n* = 7; *p* > 0.05). **(B)** Action potentials were elicited by inducing a depolarizing current of +130 pA and had a mean frequency of 20.9 ± 1.0 Hz. Application of CRH resulted in a significant increase in the frequency (23.2 ± 0.6 Hz; *n* = 8; *p* < 0.05). **(C)** Compared to control, CRH prolonged the half-width of APs from 3.0 ± 0.0 to 3.2 ± 0.0 ms (*n* = 8; *p* < 0.05). **(D)** Current-voltage relationships revealed that CRH does not change membrane resistance in CA1 pyramidal neurons. ^*^ *p* < 0.05.

Elevating Na_V_-activity enhances neuronal excitability (Clare et al., 2000) and might contribute to the CRH-mediated effect. The application of CRH did neither change the amplitude nor the voltage-dependency of sodium currents (*n* = 8; Figure 5A). CRH did not change AP amplitude (72.0 ± 3.2 vs. 65.9 ± 6.4 mV; data not shown) but slightly increased the AP frequency during current injection of +130 pA (20.9 ± 1.0 Hz control vs. 23.2 ± 0.6 Hz CRH 125 nM; *n* = 8; *p* < 0.05; Figure 5B) and increased the half-width of APs from 3.0 ± 0.0 ms under control conditions to 3.2 ± 0.0 ms (Figure 5C). Plotting membrane potentials over the corresponding current pulses demonstrates that CRH had no effect on the I–V relationship (*n* = 6; Figure 5D).

**Figure 6:**
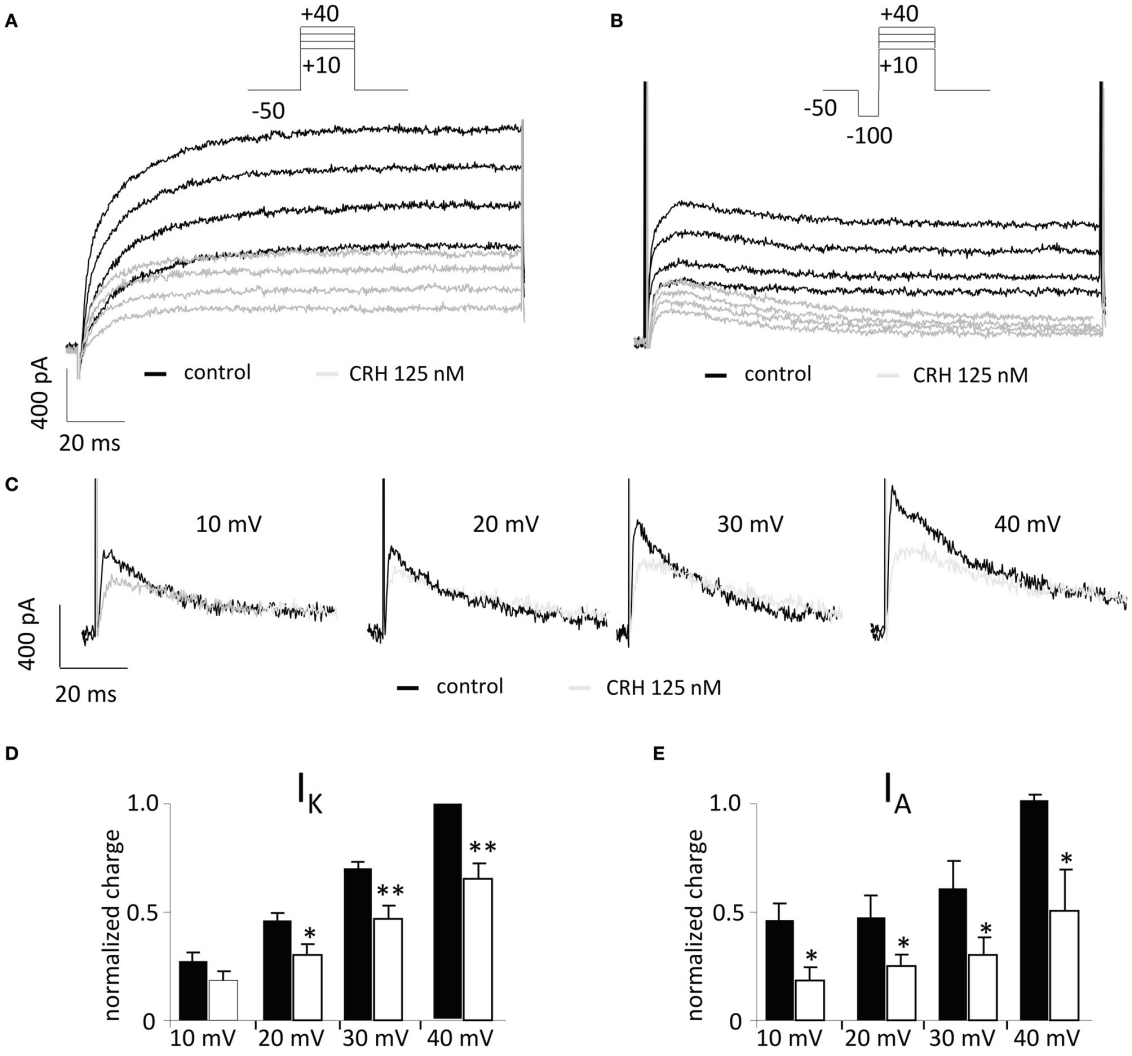
**CRH reduces currents through A-type (I_A_) and delayed rectifier (I_K_) potassium channels.** In the voltage clamp mode, currents through voltage gated potassium channels were recorded upon stepwise depolarization of CA1 pyramidal neurons from −50 to +40 mV (ΔU = 10 mV). **(A)** Representative recording traces under control conditions (black traces) and in the presence of CRH 125 nM (gray traces). **(B)** A preceding hyperpolarizing step (100 ms) de-inactivates fast I_A_ and allows the graphical subtraction of currents with and without the preceding hyperpolarizing step **(C)**, I_A_ currents can be analyzed. **(C)** Representative recording traces of I_A_ currents under control conditions (black) and in the presence of CRH 125 nM (gray). **(D)** After application of CRH, currents through delayed rectifier potassium channels (I_K_) were reduced by 14.2 ± 5.7% to 30.4 ± 6.6% dependent on the voltage step. **(E)** CRH significantly reduced I_A_ currents ranging from 30.1 ± 6.0% to 83.2 ± 26.8% depending on the voltage step (*n* = 5; *p* < 0.05). ^*^p < 0.05; ^**^p < 0.01. Insets show representative recording traces; leak subtraction was performed with the P/4 subtraction protocol.

The inhibition of potassium channels may also induce an enhancement of neuronal excitability. In the presence of CRH (125 nM) analysed charge values were reduced for I_K_ (by 15.4 ± 4.6% and by 34.6 ± 7.3% when depolarized to 20 and 40 mV, respectively; *n* = 7; Figure 6D) and for I_A_ currents (by 27.6 ± 6.0% and by 51.0 ± 19.2% at depolarization to 20 and 40 mV, respectively; *n* = 7, *p* < 0.05; Figure 6E). Depression of potassium channels did not change resting membrane potential (60.9 ± 2.0 vs. 58.8 ± 1.1 mV; *p* > 0.05) of the recorded neurons.

## Discussion

Here we provide evidence that CRH increases neuronal excitability of CA1 pyramidal neurons not by affecting synaptic transmission and mechanisms important for LTP, but by the modulation of somatic voltage-gated ionic currents important for the generation of APs. We addressed the underlying mechanism by a pharmacological approach and found an involvement of PP-2B and the potassium currents I_A_ and I_K_. Experiments in transgenic animals revealed that the CRH-mediated effect depends solely on activation of CRHR1 expressed on glutamatergic neurons.

The effect of CRH on neuronal excitability in the hippocampus is still unclear. It has been described that CRH (125–250 nM) increases neuronal excitability (Aldenhoff et al., 1983; Hollrigel et al., 1998; Wang et al., 1998; Sillaber et al., 2002) and enhances activity propagation through the hippocampal formation (von Wolff et al., 2011). In contrast, the application of either lower or higher CRH concentrations resulted in a depression of neuronal activity (Rebaudo et al., 2001). In our experiments we exclusively delivered a CRH concentration of 125 nM and observed enhanced neuronal excitability, in accordance with other studies (Smith and Dudek, 1994; Hollrigel et al., 1998; Blank et al., 2002; Eckart et al., 2002). The G-protein coupled CRHR1 is expressed in the CA1 region (Dautzenberg and Hauger, 2002) and the amount of total expression changes during development (Avishai-Eliner et al., 1996). It has been shown that the effect of CRH on PS amplitude in the hippocampal CA1 region is dependent on CRHR1 activation (Sillaber et al., 2002), but it is still unclear, whether it is mediated by CRHR1 expressed on glutamatergic or GABAergic neurons. Even though, a recent study proved CRHR1 expression in the hippocampus only in glutamatergic neurons and not in GABAergic interneurons (Refojo et al., 2011), there is no functional evidence for the generation of PS supporting this on the monosynaptic level of CA3-CA1 synapses. Our results from transgenic *CRHR*1^Glu-CKO^ and *CRHR*1^GABA-CKO^ *mice* clearly demonstrate that the CRH-mediated increase in PS amplitude is solely dependent on CRHR1 expressed on glutamatergic neurons.

CA1 stratum radiatum fEPSPs evoked by electrical stimulation of Schaffer-collaterals reflect processes of synaptic transmission that are below the threshold of AP generation in most of the target cells (Andersen et al., 1966). Whereas CRH increased PS amplitudes, fEPSPs were unaffected, making a direct CRH-dependent modulation of synaptic transmission unlikely. Electrical stimulation of the somatic region of CA3 pyramidal neurons ensures the generation of APs at the axon hillock that propagate along the Schaffer-collaterals finally resulting in transmitter release in CA1 (Meeks and Mennerick, 2007). Under these conditions, CRH significantly increased fEPSP amplitudes in the CA1 region. In contrast, when bypassing the AP generation site by a direct SC stimulation, somatic excitability does not determine fEPSP amplitudes and CRH did not affect fEPSP. Equivalent results were obtained, when testing how CRH influences CA1-LTP. LTP is the major *in vitro* correlate for processes related to memory generation (Bliss and Collingridge, 1993). Analogous to the increase of the PS amplitude, LTP was enhanced by CRH only when HFS was delivered to the CA3 pyramidal layer and remained unchanged when SCs were stimulated.

The CRH-mediated increase in neuronal excitability has been shown to be dependent on PKA and PKC (Blank et al., 2003). However, our results suggest a contribution of PP-2B in the CRH-induced enhancement of PS amplitude. It has been shown, that, dependent on the mouse strain, signal processing of CRH can be mediated by different transduction pathways. Blank et al. (2003) described that CRH activates PKA in C57/BL6 whereas in BALB/c mice CRHR1 is coupled to the PKC pathway (Blank et al., 2003). These data might explain the discrepancy to the present study, since we used for our experiments slices from CD-1 mice. To our knowledge this is the first report demonstrating an effect of CRHR1 via neuronal PP-2B. Recently, it has been shown that CRHR1 is coupled to the PP-2B-nuclear factor of activated T-cells (NFAT) signaling pathway and its activation is a prerequisite for CRH-induced catecholamine production from chromaffin cells (Dermitzaki et al., 2012). Other neuropeptides appear to affect the PP-2B/NFAT pathway including substance P (Seybold et al., 2006) and angiotensin (saygili et al., 2009).

In rat and mouse corticotrophs, CRH has been reported to stimulate Ca^2+^ entry through VGCCs in a cAMP- and PKA-dependent manner (Lee and Tse, 1997; Kovalovsky et al., 2002). However, the CRH effect in our experiments was not dependent on PKA. CRH is known to increase cellular cAMP levels by an activation of adenylyl cyclase (Chen et al., 1986; Riegel and Williams, 2008). A direct activation of PP-2B by elevated intracellular cAMP has been reported in hepatocytes (Webster et al., 2002) and PP-2B might therefore also be cAMP-sensitive in hippocampal neurons.

In voltage-clamp experiments CRH did not affect charge transfer or activation kinetics of sodium channel currents which makes a direct modulation of sodium channels unlikely. Instead, CRH increased the frequency and prolonged the decay time of APs recorded from CA1 pyramidal neurons. These results point to a modulation of ionic conductances attributed to membrane repolarization. A comparable broadening of APs was reported, when the potassium channel antagonist 4-AP was applied to hippocampal CA3 neurons (Mitterdorfer and Bean, 2002). Potassium channels from the Kv1-family are generally sensitive to 4-AP (Coetzee et al., 1999), are expressed in the hippocampus (Grosse et al., 2000) and can produce I_K_ or I_A_ depending on subunit composition (Clark et al., 2009). We clearly show that CRH depresses I_A_ and I_K_. Moreover, CRH failed to increase PS amplitudes in the presence of 4-AP that blocks K_V_1 and K_V_3 at micromolar concentrations (Johnston et al., 2010) as used in our study. These results strongly suggest that a modulation of potassium channel currents, specifically I_A_ and I_K_ is a key mechanism of the CRH-mediated enhancement of neuronal excitability of CA1 pyramidal neurons.

Potassium channels of the K_V_1 subfamily are of particular interest as a possible target for CRHR1-dependent signaling: K_V_1 are highly expressed at the axonal hillock and may influence spike initiation (Clark et al., 2009). This is well in accordance with our finding, that CRH increases neuronal excitability when transmitter release is dependent on AP initiation at CA3 pyramidal neurons and did not change synaptic activity when the site of AP initiation was bypassed. The threshold for AP generation is dynamically regulated by K_V_1 channels and inhibition increases neuronal firing (Chi and Nicol, 2007). Kv1 channels can be inactivated by PP-2B dephosphorylation (Roeper et al., 1997) so that a CRH-induced activation of PP-2B might downregulate I_K_ and I_A_.

In the presence of the L- and T-Type calcium channel antagonists nifedipine and Ni^2+^ CRH failed to increase PS amplitudes suggesting an involvement of these VGCCs. Two L-type isoforms are generally prevalent throughout the CNS: Cav1.2 and Cav1.3. In the neocortex and hippocampus, the vast majority belong to the Cav1.2 subtype (Lacinova et al., 2008). As we did not observe a reduced effect of CRH on PS amplitude in Ca_V_1.2 knockout animals, the action of CRH on PS amplitude seems to be dependent on Ca_V_1.3. Additionally, T-type channels seem to be involved as application of Ni^2+^ strongly reduced the CRH-mediated effect. However, in single cell recordings, CRH showed no impact on currents through T- or L-type calcium channels. Furthermore, blocking intracellular Ca^2+^ with BAPTA did not deteriorate the CRH-mediated effect on PS amplitude. All together, these results indicate that Ca^2+^ as a messenger is not crucial involved in the CRH-dependent increase of neuronal activity. But still, an important question remains: How are T- and L-type VGCCs involved in the observed effects? One parsimonious explanation might be that these channels contribute to the PS enlargement not by an increased Ca^2+^ current through each single channel but by an increment of the total number of channel activated since more and more neurons being faster depolarized due to the threshold lowering for AP generation triggered by CRH-dependent K_V_1 inhibition. According to this explanation, we observed elevated Ca^2+^ influx in CA1 neurons upon afferent stimulation after CRH application.

In summary, our data demonstrate that CRH does not affect synaptic excitability *per se*, but increases PS amplitude in CA1 neurons. We provide evidence that signal processing of CRH in the hippocampus of CD1 mouse strains was mediated through a PP-2B dependent signal transduction, finally attenuating somatic I_K_ and I_A_. The somatic AP initiation site appears to be essential for the CRHR1-mediated enhancement of neuronal excitability.

Hypersecretion of central CRH is thought to play an important role in the pathophysiology of stress-related mental disorders, such as major depressive disorder and PTSD. These findings may represent an important step toward understanding the cellular and molecular processes underlying CRH-mediated physiological and pathophysiological effects.

### Conflict of interest statement

The authors declare that the research was conducted in the absence of any commercial or financial relationships that could be construed as a potential conflict of interest.

## Abbreviations

CRH: corticotropin-releasing hormone
CRHR: corticotropin-releasing hormone receptor
fEPSPs: field excitatory postsynaptic potentials
HFS: high frequency stimulation
LTP: long-term potentiation
PS: population spike
SC: schaffer-collaterals
VGCC: voltage gated calcium channels.
